# Bioclinical Test to Predict Nephropathia Epidemica Severity at Hospital Admission

**DOI:** 10.3201/eid2406.172160

**Published:** 2018-06

**Authors:** Maxime Hentzien, Stéphanie Mestrallet, Pascale Halin, Laure-Anne Pannet, Delphine Lebrun, Moustapha Dramé, Firouzé Bani-Sadr, Jean-Marc Galempoix, Christophe Strady, Jean-Marc Reynes, Christian Penalba, Amélie Servettaz

**Affiliations:** University of Reims Champagne-Ardenne, Reims, France (M. Hentzien, M. Dramé);; Hôpital Universitaire Robert Debré, Reims (M. Hentzien, M. Dramé, F. Bani-Sadr, A. Servettaz);; Manchester Hospital, Charleville-Mézières, France (S. Mestrallet, P. Halin, L.-A. Pannet, D. Lebrun, J.-M. Galempoix, C. Penalba);; Groupe Courlancy, Reims (C. Strady); Institut Pasteur, Lyon, France (J.-M. Reynes)

**Keywords:** hantavirus, acute kidney injury, Puumala virus, prognosis, France, Europe, cohort studies, nephropathia epidemica, hospital admission, hemorrhagic disease, risk factors, thrombocytopenia, leukocytosis, nephrotoxic drug exposure, visual disorder, hematuria, disease severity, viruses

## Abstract

We conducted a multicenter, retrospective cohort study of hospitalized patients with serologically proven nephropathia epidemica (NE) living in Ardennes Department, France, during 2000–2014 to develop a bioclinical test predictive of severe disease. Among 205 patients, 45 (22.0%) had severe NE. We found the following factors predictive of severe NE: nephrotoxic drug exposure (p = 0.005, point value 10); visual disorders (p = 0.02, point value 8); microscopic or macroscopic hematuria (p = 0.04, point value 7); leukocyte count >10 × 10^9 ^cells/L (p = 0.01, point value 9); and thrombocytopenia <90 × 10^9^/L (p = 0.003, point value 11). When point values for each factor were summed, we found a score of <10 identified low-risk patients (3.3% had severe disease), and a score >20 identified high-risk patients (45.3% had severe disease). If validated in future studies, this test could be used to stratify patients by severity in research studies and in clinical practice.

## Abstract 2

This test identifies patients at low, intermediate, and high risk for severe disease.

Hantaviruses (family *Bunyaviridae*, genus *Hantavirus*) are enveloped viruses with negative, trisegmented, single-stranded RNA genomes that can induce hemorrhagic fever with renal syndrome (HFRS) or hantavirus pulmonary syndrome (*1*). The viruses that cause HFRS include Hantaan virus, Dobrava-Belgrade virus, Seoul virus, Tula virus, and Puumala virus (PUUV). PUUV, predominantly transmitted by the bank vole (*Myodes glareolus*), is the most common species of hantavirus in central and north Europe and frequently causes a mild form of HFRS, nephropathia epidemica (NE) (*1*–*3*). NE is endemic in the northeast of France; the Champagne-Ardenne and Picardie regions are most affected. In 2015, the incidence was 12.02 cases/100,000 inhabitants in the Ardennes Department (*4*).

The clinical presentation of NE is the same throughout Europe (*5*); typically, the signs and symptoms of NE are sudden onset high-grade fever, headache, visual disorders, gastrointestinal irregularities, and low back pain. The biological profile of NE is characterized by acute kidney injury (AKI) associated with proteinuria, thrombocytopenia, and biological inflammatory syndrome, including elevated leukocyte count and C-reactive protein level (*3*).

NE is a benign disease with a low case-fatality rate (<1%) (*3*,*6*) and favorable early and long-term outcomes (*7*–*9*). Nevertheless, patients with mild disease are frequently held in hospitals for continued renal observation and treatment. Moreover, a severe form of the disease can develop, although infrequently, additionally contributing to the high frequency of prolonged hospitalization for this disease (*10*,*11*). Severe forms of NE have been defined according to varying criteria in the literature but are generally defined by using AKI severity criteria (*11*–*14*), such as the RIFLE (risk for renal dysfunction, kidney injury, failure or loss of kidney function, and end-stage renal disease) (*15*), KDIGO (Kidney Disease: Improving Global Outcomes) (*16*), or AKIN (Acute Kidney Injury Network) (*17*) classifications. Renal replacement therapy is required in ≈5% of patients with NE (*3*).

The predictive factors of severe NE are not well known, and consequently, identifying the patients at low and high risk for severe NE is not possible. Patients are, therefore, frequently kept hospitalized for observation (*10*,*11*). Improved knowledge of the predictors of severe forms could help in the identification of patients at low and high risk for severe NE in routine care and, therefore, reduce the prolonged hospitalization of patients at low risk. The main objective of this study was to identify the predictive factors for severe disease among patients with serologically proven NE in the Champagne-Ardenne region in France and to derive a bioclinical score that enables identification of patients more likely to develop severe NE.

## Materials and Methods

### Study Design and Patients

In this multicenter, retrospective cohort study, we included all patients living in Ardennes Department, France, who were hospitalized for serologically proven NE during January 2000–December 2014. We identified hospitalized patients fulfilling the inclusion criteria by searching through microbiology laboratory databases of the following centers: Reims University Hospital (Reims, France); Charleville-Mézières Hospital (Charleville-Mézières, France); Sedan Hospital (Sedan, France); and the National Reference Center for Hantaviruses (Lyon, France). We included only patients positive for hantavirus IgM and IgG by the Hantavirus IgM and IgG DxSelect ELISA kits (Focus Diagnostics, Cypress, CA, USA) that were confirmed by the National Reference Center for Hantavirus. We excluded patients who sought treatment at an emergency department who were not admitted and patients who had a severe form (defined later) at admission. The study was conducted in accordance with French Jarde’s law on retrospective data studies and the Declaration of Helsinki.

### Data Collection and Definitions

We extracted all data retrospectively from patients’ medical records using a standardized case report form. Data were deidentified, then extracted and stored for analysis. We collected the following variables: sociodemographic data, updated Charlson comorbidity index (not adjusted for age) (*18*), center, clinical presentation, intake of nephrotoxic treatments, results of HFRS laboratory diagnosis, date of first symptoms, date and duration of hospitalization, results of standard biological data at baseline and during hospitalization, and the occurrence of severe disease.

The clinical characteristics collected at admission were fever (temperature >38°C); myalgia; low back pain; visual disorders (myopic shift, blurred vision); chest symptoms (cough, dyspnea, chest auscultation abnormalities, pathologic chest radiograph); digestive symptoms (nausea, diarrhea, abdominal pain); oliguria (urine output <500 mL/d); neurologic signs (meningism, headache without meningism); and nonsevere hemorrhagic signs (hematuria, mucosal bleeding, purpura, petechiae, conjunctival hemorrhage). We considered the following nephrotoxic drug exposures during hospitalization, including during the first 24 h: nonsteroidal antiinflammatory drugs, iodinated contrast media, diuretics, renin angiotensin aldosterone system inhibitors, and nephrotoxic antimicrobial drugs (aminoglycosides, glycopeptides). Biological data collected at admission included hemoglobin level, leukocyte count, neutrophil count, platelet count, C-reactive protein, plasma creatinine, aspartate aminotransferase, alanine aminotransferase, hematuria (>10 cells/mm^3^ in urine sediment or macroscopic hematuria), and proteinuria. If multiple plasma creatinine values were available between 2 weeks and 1 year after discharge, we also collected the first of these readings. We defined severe NE as the occurrence of >1 of the following criteria during hospitalization: hypovolemic, hemorrhagic, or septic shock; plasma creatinine level >353.6 µmol/L (*9*,*16*,*19*); anuria (urine output <300 mL/d); need for dialysis; hemorrhage requiring blood transfusion; admission to the intensive care unit; or death.

### Statistical Analysis

Quantitative variables are presented as mean ± SD or median (interquartile range [IQR]), as appropriate, and qualitative variables as number (percentage). We assessed the differences between groups using the χ^2^ test or Fisher exact test for categorical variables and Student *t*-test or the Mann-Whitney U-test for continuous variables, as appropriate.

We performed univariable and multivariable logistic regression to develop the prognostic model and generate unadjusted odds ratios (OR) and adjusted ORs (aOR) and the associated 95% CIs. Occurrence of a severe form of NE was the primary endpoint. We considered baseline characteristics potential explanatory variables and decided not to include urine dipstick proteinuria at admission as a predictor of severe NE because of the amount of missing data for this variable. We used a manual stepwise method to identify variables independently associated with the occurrence of severe NE and systematically adjusted the multivariable model for the time since symptom onset. We also performed bootstrap analysis to evaluate the internal validity of the model performance. Replication on 2,000 different samples drawn with replacement was the bootstrap method performed. We used the C statistic and the Hosmer-Lemeshow goodness-of-fit test to assess model performances (discrimination and calibration).

For the development of the bioclinical score, we assigned a point value for each independent factor according to the aOR of the final model. The aORs were multiplied by 3, rounded to the nearest integer, and then summed. We constructed a receiver operating characteristic curve and obtained C statistic 95% CIs using bootstrap methods. We performed statistical analyses with SAS version 9.4 (SAS Institute Inc., Cary, NC, USA) and considered p values <0.05 significant.

## Results

### Population Characteristics

**Figure 1:**
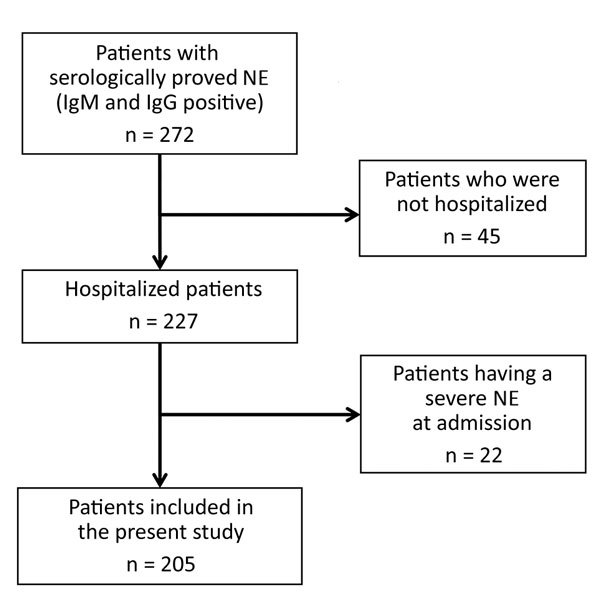
Determination of study population used to derive a bioclinical score that enables identification of patients more likely to develop severe NE. Patients were those living in Ardennes Department, France, who were hospitalized for serologically proven NE during January 2000–December 2014. NE, nephropathia epidemica.

Among the 272 patients with NE during the study period, 227 (83.5%) were hospitalized. Of these, 22 (9.7%) had a severe form of NE at admission and were excluded from the study (Figure 1). The patients with severe NE at admission were hospitalized significantly later after symptom onset (7.8 ± 3.1 days) than the patients included in the study (5.3 ± 2.7 days; p = 0.0003). These excluded patients were all men; were older than included patients; and had higher nephrotoxic drug exposure, more frequent low back pain, more frequent oliguria during the clinical course, and higher leukocyte counts than included patients (M. Hentzien, unpub. data).

Among the 205 patients included in the analysis, the mean length of hospital stay was 7.2 ± 3.5 days, their mean age was 38.6 ± 14.3 years, 74.6% were men, and the median Charlson comorbidity index was 0 (IQR 0–0) (Table 1). Only 24 (11.7%) patients had a Charlson comorbidity index >0, and 60 (29.3%) patients had taken nephrotoxic drugs, predominantly nonsteroidal antiinflammatory drugs (17.1%), around the time of admission.

**Table 1:** Clinical characteristics of 205 hospitalized patients with nephropathia epidemica, Ardennes Department, France, January 2000–December 2014*

Characteristic	Value	Missing
Sex		
M	153 (74.6)	0
F	52 (25.4)	0
Age, y, mean ± SD	38.6 ± 14.3	0
Charlson comorbidity index score, median (IQR)	0 (0–0)	0
>0	24 (11.7)	
Chronic renal disease	4 (2.0)	0
Time from onset of symptoms to hospitalization, d, mean ± SD	5.3 ± 2.7	0
Nephrotoxic drug intake†	60 (29.3)	0
Nonsteroidal anti-inflammatory drugs	35 (17.1)	0
Iodinated contrast media	9 (4.4)	0
Diuretics	14 (6.8)	0
Renin angiotensin aldosterone system inhibitors	12 (5.9)	0
Nephrotoxic antimicrobial drugs	5 (2.4)	0
Clinical signs and symptoms at admission		
Fever	203 (99.5)	1
Arthromyalgia	167 (81.5)	0
Low back pain	92 (44.9)	0
Visual disorders	68 (33.2)	0
Chest symptoms	96 (46.8)	0
Cough	65 (31.7)	0
Dyspnea	11 (5.4)	0
Chest auscultation abnormalities	34 (16.6)	0
Pathological chest radiograph	41 (23.0)	27
Digestive symptoms	144 (70.2)	0
Nausea	107 (52.2)	0
Diarrhea	38 (18.5)	0
Abdominal pain	104 (50.7)	0
Oliguria during clinical course	32 (15.6)	0
Neurologic signs	149 (72.7)	0
Meningism	21 (10.2)	0
Headache without meningism	147 (71.7)	0
Nonsevere hemorrhagic signs‡	28 (13.7)	0
Laboratory findings at admission		
Hemoglobin, g/L, mean ± SD	146 ± 15	10
Platelet count, × 10^9^/L, median (IQR)	92 (66–123)	1
>90	104 (50.9)	
50–<90	78 (38.2)	
<50	22 (10.8)	
Leukocyte count, × 10^9 ^cells/L, mean ± SD	8.0 ± 3.2	5
>10	45 (22.5)	5
Neutrophil count, × 10^9 ^cells/L, mean ± SD	5.5 ± 2.4	7
Lymphocyte count, × 10^9 ^cells/L, mean ± SD	1.3 ± 0.7	11
<1	78 (40.2)	11
Creatinine, µmol/L, median (IQR)	98 (79–130)	6
Alanine aminotransferase, × ULN, median (IQR)	1.0 (1.0–1.5)	28
Aspartate aminotransferase, × ULN, median (IQR)	1.0 (1.0–1.5)	26
C-reactive protein, median (IQR)	79 (46–117)	2
>100 mg/L, >952 nmol/L	70 (34.5)	2
Hematuria		6
Absent	103 (51.8)	
Microscopic	90 (45.2)	
Macroscopic	6 (3.0)	
Urine dipstick proteinuria§		128
0	15 (17.9)	
+	15 (17.9)	
++	15 (17.9)	
+++	20 (23.8)	
++++	12 (14.3)	

*Vales are n (%) unless otherwise specified. IQR, interquartile range; ULN, upper limit of normal. †More than 1 factor is possible. ‡Defined as hemorrhagic signs not requiring blood transfusion. §For urine dipstick readings, 0 indicates no proteinuria, + indicates ≈30 mg/dL, ++ indicates ≈100 mg/dL, +++ indicates ≈300 mg/dL, and ++++ indicates ≈2,000 mg/dL.

### Clinical Course

**Figure 2:**
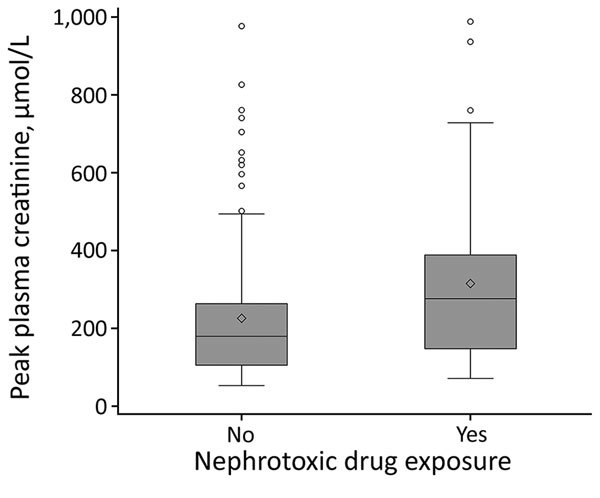
Peak creatinine plasma levels in patients hospitalized for nephropathia epidemica by nephrotoxic drug exposure, Ardennes Department, France, January 2000–December 2014. Top and bottom borders of boxes indicate interquartile ranges (IQRs), horizontal lines within boxes indicate medians, diamonds indicate means, and circles represent outliers. A whisker is drawn from the upper edge of the box to the largest observed value within the upper fence (located at 1.5 × IQR above the 75th percentile), and another is drawn from the lower edge of the box to the smallest observed value within the lower fence (located at 1.5 × IQR below the 25th percentile). Peak plasma creatinine levels were higher in the patients exposed to nephrotoxic drugs (p = 0.002).

Creatinine plasma level peaked at a median of 198 (IQR 110–318) µmol/L at a median of 8 (IQR 6–10) days after symptom onset. Proteinuria also peaked at a median of 8 (IQR 6–10) days after symptom onset at a median of 2.3 (IQR 0.9–4.9) g/d. Patients who were exposed to nephrotoxic drugs had a higher median peak creatinine plasma (277 [IQR 148–389] µmol/L) than patients who were not (180 [IQR 106–264] µmol/L; p = 0.002; reference range 53–106 µmol/L; Figure 2), although their median creatinine plasma levels at admission were similar (exposed 107 [IQR 77–161] µmol/L vs. not exposed 97 [IQR 80–123] µmol/L; p = 0.58).

### Occurrence of Severe NE

During hospitalization, NE progressed in severity in 45 (22.0%) patients. Among these patients, 41 (91.1%) had plasma creatinine >353.6 µmol/L, 6 (13.3%) had anuria, 3 (6.7%) required dialysis, 2 (4.4%) experienced shock, and 2 (4.4%) required admission to the intensive care unit. Of the 4 patients with severe NE without elevated plasma creatinine levels of >353.6 µmol/L, 2 experienced shock and 3 had >1 d of anuria. No patient had hemorrhaging requiring a blood transfusion, and no patient died.

The time from onset of symptoms to hospitalization in patients who had severe NE (6.0 ± 2.8 d) was not significantly different from those who did not (5.4 ± 2.8 d; p = 0.11), enabling baseline characteristic comparison. Patients who had severe NE had a significantly longer hospital stay (8.5 ± 3.5 d) than did patients who did not (6.9 ± 3.4 d; p = 0.0005). Among patients whose biological data was available between day 15 and 1 year after the end of the hospitalization (60.0%, n = 123), the median value of the first observed plasma creatinine level was 80 (IQR 68–87, range 40–128) µmol/L. This variable was not significantly different between patients who had severe NE (81 [IQR 76–88] µmol/L) and those who did not (78 [IQR 67–87] µmol/L; p = 0.21).

### Predictors of Severe NE and Derived Predictive Score

By univariable analysis (Table 2), the factors significantly associated with the occurrence of severe NE were nephrotoxic drug intake, visual disorders, hematuria, leukocyte count >10 × 10^9 ^cells/L, C-reactive protein >100 mg/L (>952 nmol/L), and thrombocytopenia <90 × 10^9^/L. By multivariable analyses (Table 2), the factors that remained significantly associated with the occurrence of severe NE after adjustment were nephrotoxic drug intake (aOR 3.25, 95% CI 1.42–7.46; p = 0.005), visual disorders (aOR 2.64, 95% CI 1.17–5.96; p = 0.02), microscopic or macroscopic hematuria (aOR 2.37, 95% CI 1.03–5.43; p = 0.04), leukocyte count >10 × 10^9 ^cells/L (aOR 3.03, 95% CI 1.25–7.39; p = 0.01), and thrombocytopenia <90 × 10^9^/L (aOR 3.74, 95% CI 1.59–8.81; p = 0.003). Because of the collinearity between the leukocyte count and C-reactive protein level and the fact that the predictive ability of the leukocyte count was better, we decided to include only the leukocyte count in the score. ORs obtained by bootstrap analysis of the final multivariable model were similar to those in the final multivariate model, suggesting good internal validity.

**Table 2:** Univariable and multivariable analysis of factors predictive of severe nephropathia epidemica, Ardennes Department, France, January 2000–December 2014*

Category	Univariable analysis, n = 205				Multivariable analysis,† n = 194	
OR‡ (95% CI)	p value	Missing	aOR (95% CI)	p value
Age >40 y	0.63 (0.31–1.27)	0.19	0			
Female sex	0.80 (0.37–1.76)	0.58	0			
Charlson comorbidity index score >1	0.68 (0.22–2.11)	0.51	0			
Nephrotoxic drug intake	2.12 (1.06–4.23)	0.03	0		3.25 (1.42–7.46)	0.005
Chronic renal disease	1.19 (0.12–11.72)	0.88	0			
Low back pain	1.38 (0.71–2.68)	0.34	0			
Visual disorders	3.01 (1.53–5.20)	0.002	0		2.64 (1.17–5.96)	0.02
Microscopic or macroscopic hematuria	2.54 (1.26–5.11)	0.009	6		2.37 (1.03–5.43)	0.04
Platelet count <90 × 10^9^/L	3.15 (1.53–6.46)	0.002	1		3.74 (1.59–8.81)	0.003
Leukocyte count >10 × 10^9 ^cells/L	2.87 (1.36–6.05)	0.006	5		3.03 (1.25–7.39)	0.01
C-reactive protein >100 mg/L, >952 nmol/L	2.19 (1.12–4.31)	0.02	2			
Alanine aminotransferase >1 × ULN	1.66 (0.76–3.59)	0.20	28			
Aspartate aminotransferase >1 × ULN	1.18 (0.53–2.61)	0.69	26			

*aOR, adjusted odds ratio; OR, odds ratio; ULN, upper limit of normal. †The multivariable model was systematically adjusted for the time between onset of symptoms and hospitalization. The C statistic for the multivariable model was 0.81. Hosmer-Lemeshow goodness-of-fit test: p = 0.87. ‡Logarithm for odds of severe nephropathia epidemica = −3.0830 − (0.1214 × days between first symptoms and hospitalization) + (0.9693 × visual disorder) + (0.8621 × hematuria) + (1.1099 × elevated leukocyte count) + (1.3185 × thrombocytopenia) + (1.1787 × nephrotoxic drug exposure). §Nephrotoxic drugs included nonsteroidal antiinflammatory drugs, iodinated contrast media, diuretics, renin angiotensin aldosterone system inhibitors, and nephrotoxic antimicrobial drugs (aminoglycosides, glycopeptides).

**Figure 3:**
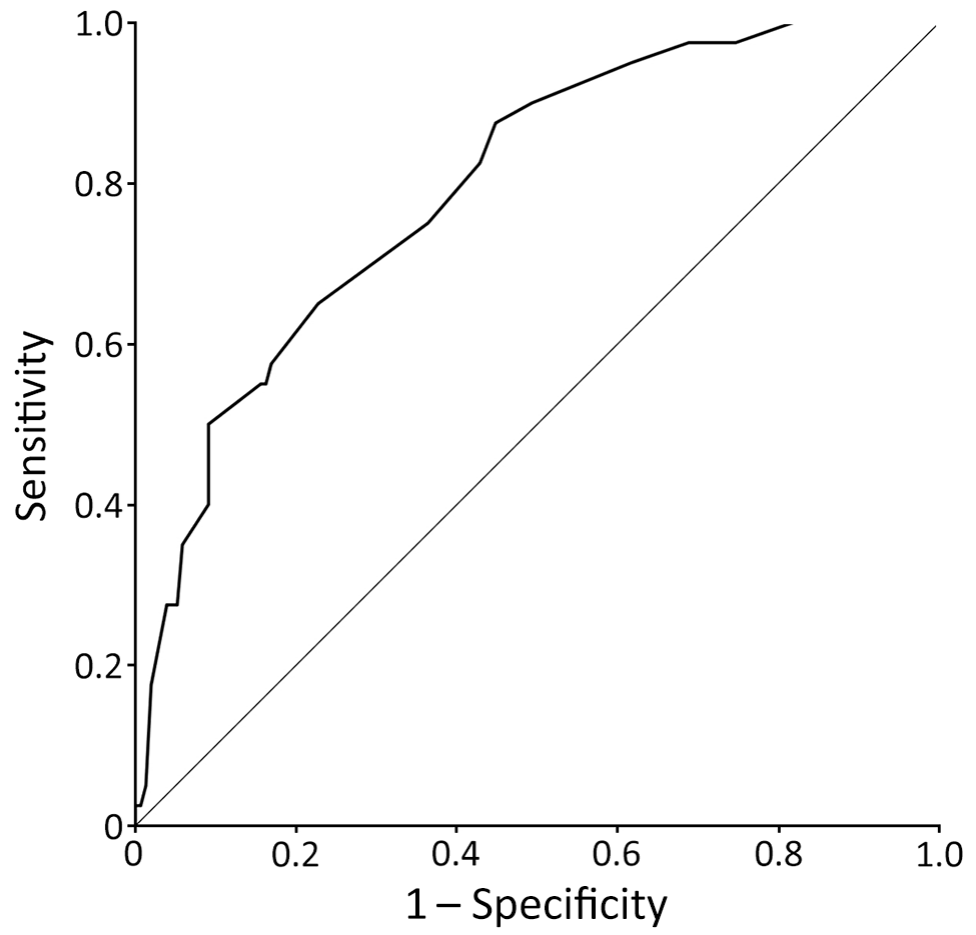
Receiver operating characteristic curve of test to predict development of severe nephropathia epidemica among patients hospitalized for nephropathia epidemica, Ardennes Department, January 2000–December 2014. Severe nephropathia epidemica was defined as the occurrence of >1 of the following criteria: hypovolemic, hemorrhagic, or septic shock; plasma creatinine level >353.6 µmol/L; anuria (urine output <300 mL/d); acute kidney injury or hydroelectrolytic disorders requiring dialysis; hemorrhage requiring blood transfusion; admission to the intensive care unit; or death. Area under the curve is 0.80.

With this statistical analysis, we derived a test to predict the occurrence of severe NE. In this test, point values for each of the predictive factors we identified (Table 3) are added up for a possible total of 45, with 0 indicating the lowest risk and 45 indicating the highest. The mean observed score for our patient population was 16.4 ± 10.0 (range 0–45). The uncorrected C statistic of the receiver operating characteristic curve for our test (Figure 3) was the same as the C statistic obtained from bootstrap analysis (0.80, 95% CI 0.72–0.87). Using this test for our patient population, we found that a score of ≤10 identified patients at low risk for severe NE (3.3% of patients in this group had severe disease) and a score ≥20 identified patients at high risk for severe NE (45.3% of patients in this group had severe disease) (Table 4).

**Table 3:** Point value assigned for each predictive factor of severe nephropathia epidemica to be used for bioclinical assay to access risk for nephropathia epidemica severity*

Predictive factor	Point value
Hematuria	7
Visual disorders	8
Leukocyte count >10 × 10^9 ^cells/L	9
Nephrotoxic drug exposure†	10
Thrombocytopenia <90 × 10^9^/L	11

*Predictive factors were assessed at admission. †Nephrotoxic drugs included nonsteroidal antiinflammatory drugs, iodinated contrast media, diuretics, renin angiotensin aldosterone system inhibitors, and nephrotoxic antimicrobial drugs (aminoglycosides, glycopeptides).

**Table 4:** Events observed in patients hospitalized with NE by risk group as determined by bioclinical test score, Ardennes Department, France, January 2000–December 2014*

Risk group	Score	No. NE patients	Observed severe NE, no. (%)
Low	0–10	61	2 (3.3)
Intermediate	11–19	80	14 (17.5)
High	20–45	53	24 (45.3)

*Data were missing for 11 patients, who were excluded in this analysis. However, similar results for observed severe NE were obtained when assuming a score of zero for missing data: low (4.7%, 3/64), intermediate (20.1%, 18/87), and high (44.4%, 24/54). The leukocyte count and platelet count at admission were missing from the 1 additional patient with severe NE categorized as low risk in this analysis. NE, nephropathia epidemica.

## Discussion

In this study, which included a large representative cohort of patients hospitalized for NE during January 2000–December 2014, we identified nephrotoxic drug intake, visual disorder, microscopic or macroscopic hematuria, leukocyte count >10 × 10^9 ^cells/L, and thrombocytopenia (<90 × 10^9^/L) as independent predictive factors of severe NE. We derived a simple bioclinical test that can be calculated on the day of admission that makes it possible for clinicians to distinguish between patients at low, intermediate, and high risk of developing severe NE.

Our patients were comparable to patients described in other studies. Infected patients were young (*9*,*19*), predominantly men (*9*,*11*,*19*–*21*), with no or few concurrent conditions (*9*,*11*,*19*). The clinical presentation was also similar to other reports (*11*). The time elapsed between first symptoms and hospitalization (≈5 days) was the same as that reported in the literature (*9*,*11*,*20*,*22*). The clinical course observed in our patients was classical, with a peak of plasma creatinine and proteinuria around 8 days after onset of symptoms (*9*,*21*) and a mean duration of hospitalization of 7 days (*11*).

According to the definition we used, severe NE developed during hospitalization in 22% of patients, and 9.7% of patients had a severe form at admission. The total number of severe forms we observed was similar to that found by Outinen et al. (34%), who used similar severity criteria (but excluded anuria, hemorrhage requiring blood transfusion, admission to the intensive care unit, and death from their definition) (*9*). Median peak of plasma creatinine was similar and renal replacement therapy was low, as in other studies (*9*,*11*).

Contrary to other studies (*11*–*14*), we decided not to use strict RIFLE (*15*), KDIGO (*16*), or AKIN criteria (*17*) to define severe NE because, in our experience, these criteria are too sensitive yet not specific enough to reflect severe NE. In particular, a 3-fold increase in plasma creatinine over baseline does not seem adequate for this disease. First, in this young population with few, if any, concurrent conditions, baseline plasma creatinine level is rarely available in clinical practice (*9*). Second, in this population, a 3-fold increase would include patients with a peak creatinine plasma level <200–300 µmol/L, which is very common in NE (*13*). Consequently, severe forms might be overrepresented in studies involving such criteria. By this definition, severe NE could represent as many as 65% of the total cases during the course of disease (*13*), whereas NE is usually a benign disease with favorable short- and long-term outcomes. In fact, a reversible form of AKI is frequent, and severe complications are rare.

Most severe NE patients had plasma creatinine >353.6 µmol/L (as in our definition of severe disease), which is expected according to the bioclinical course of the disease. Elevations in plasma creatinine are linked to other criteria, such as anuria and the need for dialysis, which reflect the severity of AKI and generally the severity of NE. However, some defining elements of severe NE, such as death, shock, hemorrhage requiring transfusion, or admission to the intensive care unit, are not necessarily linked to AKI. In our study, a nonnegligible portion (9%) of severe NE forms were not related to plasma creatinine elevations; not classifying the patients with these disease forms as severe would have been damaging to the validity of this study, as these patients were in need of urgent inpatient care.

All of the factors we found independently predictive of severe NE have already been mentioned in the literature. Nephrotoxic drug intake was frequent in our study; about one third of patients had taken this type of drug, probably to treat the pain and fever that are common in NE. Nephrotoxic drug use is common and should be systematically recorded at initial examination and considered when assessing the risk for severe NE. In 1 study, patients exposed to ibuprofen or diclofenac were found to have higher initial and peak creatinine levels, even after adjustment for confounders (*23*). Physicians should be aware of this association when assessing patients with possible NE and avoid administering nephrotoxic drugs to these patients because often other drugs may be administered in their place, potentially modifying patient outcomes for the better.

We found visual disorders in 68 of the 205 patients in our cohort, and this variable was found to be independently associated with the occurrence of severe NE. The association between visual disorders and NE severity has previously been investigated, with conflicting results. Hautala et al. found an association between the change in anterior chamber depth and creatinine plasma level (*24*). Conversely, Theiler et al. failed to find any significant association between blurred vision or myopic shift, as assessed by an ophthalmologist, and NE severity, although the number of patients included was low (n = 18) (*25*). Other studies in which patients were examined by an ophthalmologist (*25*,*26*) showed increased visual disorders (e.g., blurred vision, myopic shift) compared with other studies, probably because of selection bias or higher sensitivity of the ophthalmologic examination. In this study, we found a strong association between patient’s reported ocular involvement and the risk for severe NE, probably because only symptomatic ocular disorders, which are the most severe, were taken into account. Loss of visual acuity in patients with NE might reflect higher tissue permeability, which could explain an association with greater renal damage (*26*).

Hematuria is frequently observed in NE. We found a prevalence of 50% hematuria at admission, whereas other studies have reported prevalences of 25%–58% (*11*,*23*,*27*). At least 1 study found an association between hematuria (but not thrombocytopenia) and progression to severe AKI (*28*). Hematuria is also considered a marker of NE severity predictive of polyuria (*29*). In Hantaan virus infections, hematuria has been associated with the occurrence of severe HFRS (*30*,*31*). Leukocyte count has previously been identified as a predictive marker of severe NE (*32*) and a predictive marker of death from HFRS among populations in China, where Hantaan virus and Seoul virus are the 2 major circulating species (*22*,*30*,*33*,*34*). Leukocyte count was collinear with C-reactive protein level, which has also been found associated with severe NE (*13*,*35*). Thrombocytopenia ≤90 × 10^9^/L has been found associated with a more severe course of disease (*13*,*27*,*31*,*34*,*36*,*37*).

Proteinuria, especially urine sample proteinuria:creatinuria ratio, or dipstick proteinuria at admission would have been good candidate predictors of severe NE (*12*,*13*,*38*,*39*), as would have been urine output during the first 24 h and data on tobacco use (*19*). However, because of the retrospective design, these factors were not reliably assessed on admission in our centers, contrary to during follow-up. Another good candidate predictor might be albumin level (or proteinemia) at admission, as this parameter could reflect severe vascular leakage or an increased degree of systemic inflammation (*9*). Unfortunately, albumin level was not routinely assessed on admission in our centers. Other independent risk factors, such as elevated urokinase-type plasminogen activator receptor plasma level, interleukin 6, pentraxin-3, indoleamine 2,3-dioxygenase, cell-free DNA, Mac-2 binding protein, cerebrospinal fluid neopterin concentration, and urine GATA-3 mRNA level, have been reported in the literature (*14*,*32*,*35*,*40*–*42*). However, tests of these parameters are not typically available in clinical practice.

The simple test proposed here could be calculated on admission to evaluate the risk for severe NE (using the more stringent definition of severe NE) and is applicable for patients for whom the need for hospitalization is being considered. This bioclinical test could help physicians avoid prolonged hospitalizations of low-risk patients and better treat high-risk patients, keeping them hospitalized and monitored. The discriminatory ability of the test score was satisfactory, with an area under the curve (C statistic) of 0.80. The model also showed good internal validity, as parameter estimates were stable after bootstrapping. In another study, a predictive test was proposed to identify patients at high risk for severe AKI in acute NE (*13*). In a retrospective study in Germany, Latus et al. studied 137 patients who had normal kidney function at hospital admission during 2001–2012 and identified 3 predictive factors (thrombocytopenia, proteinuria, 12-fold elevated C-reactive protein) of severe AKI (defined as kidney injury and failure of kidney function, according to the RIFLE criteria) (*15*). Patients without these factors had a relatively high probability (18%) of developing severe AKI, as defined by the authors, probably because of the definition used to characterize the severe form.

Our study has several limitations that should be acknowledged. Atypical NE cases might have been underdiagnosed, especially in younger patients with mild disease. However, these patients might not be those who would most benefit from the use of the test we developed. In addition, the retrospective design of our study incurs a high risk for misclassification and missing data. Also, our study was not performed nationwide, although it was conducted in the more NE-endemic regions and in centers experienced with treating NE. Finally, external validation of our test is needed before recommending wider use. Despite these limitations, we believe that the bioclinical test proposed could be helpful in the initial evaluation of patients and subsequent management of NE, given that this test is easy to use in routine practice. This scoring system could also be useful in clinical research, allowing for stratification and evaluation of patients by risk group.

In conclusion, we developed a simple bioclinical test assessing the presence of visual disorders, nephrotoxic drug exposure, leukocytosis, hematuria, and thrombocytopenia at hospital admission to discriminate patients at low, intermediate, and high risk for severe NE. This test could be helpful in identifying patients at high risk for severe NE in clinical practice, pending external validation with other potentially larger-scale studies.
